# Comparative evaluation of non-contrast MRI versus gadoxetic acid-enhanced abbreviated protocols in detecting colorectal liver metastases

**DOI:** 10.1186/s13244-024-01886-3

**Published:** 2025-01-02

**Authors:** Haoran Dai, Cheng Yan, Xi Jia, Yuyao Xiao, Xinyue Liang, Chun Yang, Kai Liu, Mengsu Zeng

**Affiliations:** 1 School of Health Science and Engineering, University of Shanghai For Science and Technolgy, Shanghai, China; 2 Department of Radiology, Zhongshan Hospital, Fudan University, Shanghai, China; 3 Shanghai Institute of Medical Imaging, Shanghai, China

**Keywords:** Non-contrast MRI, Gadoxetic acid-enhanced MRI, Colorectal liver metastases, Abbreviated protocols, Diagnostic efficacy

## Abstract

**Purpose:**

This study compares the diagnostic efficacy of non-contrast abbreviated MRI protocols with Gadoxetic acid-enhanced abbreviated MRI for detecting colorectal liver metastasis (CRLM), focusing on lesion characterization and surveillance.

**Methods:**

Ninety-four patients, including 55 with pathologically verified CRLM, were enrolled, totaling 422 lesions (287 metastatic, 135 benign). Two independent readers assessed three MRI protocols per patient: Protocol 1 included non-contrast sequences (T2-weighted turbo spin-echo, T1-weighted Dixon, diffusion-weighted imaging (DWI), and ADC mapping). Protocol 2 included gadoxetic acid enhancement with hepatobiliary phase imaging, T2 TSE, DWI, and ADC maps. Protocol 3 utilized the standard Gadoxetic Acid-enhanced MRI sequence, which included pre-contrast T1-weighted imaging, T1-weighted Dixon sequences, post-contrast T1-weighted imaging (including arterial, portal venous, transitional and hepatobiliary phases), and additional T2-weighted and DWI sequences. Diagnoses were scored on a 5-point scale (benign = 1; malignant = 5), with scores ≥ 3 indicating CRLM. ROC curves analyzed diagnostic accuracy, comparing area under the curve (AUC) values across protocols.

**Results:**

No significant difference in AUCs was observed between Protocol 1 (0.899–0.909) and Protocol 2 (0.906–0.931) versus Protocol 3 (0.935–0.939) (*p* = 0.091–0.195). For lesions ≤ 10 mm, Protocol 1 was slightly inferior to Protocol 3 (*p* = 0.002–0.032), while Protocol 2 remained comparably effective (*p* = 0.096–0.179). These findings held when using a threshold of ≥ 4 to define CRLM.

**Conclusion:**

The non-enhanced abbreviated MRI protocol is as effective as the gadoxetic acid-enhanced protocol in identifying CRLM. The proposed Ab-MRI approach may be a viable alternative for CRLM surveillance.

**Critical relevance statement:**

The non-enhanced abbreviated MRI (Ab-MRI) protocol is as effective as the gadoxetic acid-enhanced protocol in identifying colorectal liver metastasis (CRLM). The proposed Ab-MRI approach may be a viable alternative for CRLM surveillance.

**Key Points:**

- Two abbreviated protocols are proposed for colorectal liver metastasis (CRLM) surveillance.
- The non-enhanced protocol showed equivalent efficacy and was more cost-effective.
- The non-enhanced protocol may be a viable alternative for CRLM surveillance.

**Graphical Abstract:**

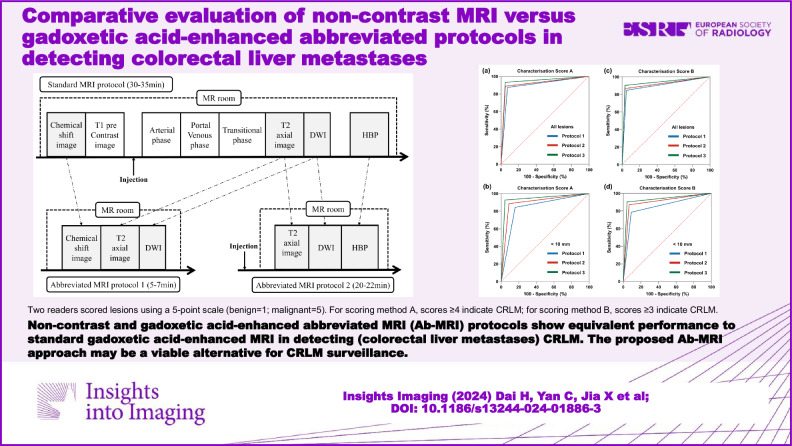

## Introduction

Colorectal cancer (CRC) is a significant global health issue, characterized by high incidence and mortality rates [1, 2]. Approximately half of CRC patients develop liver metastases (CRLM), which significantly worsen prognosis and quality of life [3–5]. Early detection and continuous monitoring of CRLM are crucial for optimizing treatment strategies, assessing therapeutic efficacy, and predicting patient outcomes [6, 7].

Conventional monitoring methods, such as computed tomography (CT) scans and standard magnetic resonance imaging (MRI), have been effective in diagnosing liver metastases from colorectal cancer [8, 9]. However, these methods often come with drawbacks, including radiation exposure, high expense, and long examination times [10, 11]. Contrast-enhanced MRI (CE-MRI) is a powerful tool but carries the risk of adverse reactions to contrast agents. Advances in medical technology have facilitated the investigation of non-contrast MRI and streamlined the use of gadolinium-based contrast agents aiming to reduce invasiveness while maintaining diagnostic accuracy [12–15].

Recent studies have shown that abbreviated MRI (Ab-MRI) protocols, both with and without contrast agents, can achieve comparable diagnostic performance to full CE-MRI protocols [16–18]. These protocols are particularly valuable in reducing examination time and cost, making them more accessible for routine clinical use [19, 20]. In this study, we compare the diagnostic efficacy of non-contrast abbreviated MRI protocols with gadoxetic acid-enhanced abbreviated MRI for detecting CRLM, focusing on lesion characterization and surveillance efficiency. Our goal is to determine whether a non-enhanced abbreviated MRI protocol can serve as a viable and cost-effective alternative to traditional CE-MRI protocols.

## Materials and methods

### Patients

This retrospective study was approved by the Institutional Review Board of Zhongshan Hospital and was conducted in accordance with the Declaration of Helsinki. In accordance with the retrospective nature of the study, the requirement for written informed consent was waived.

**Fig. 1:**
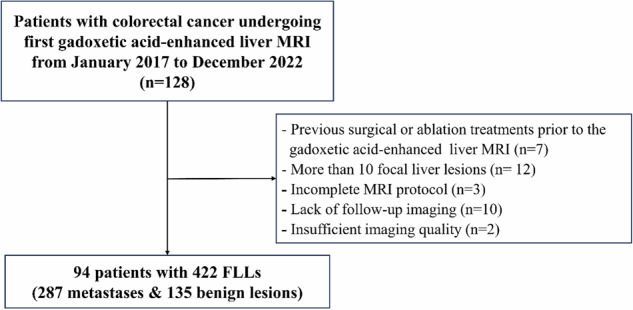
Flowchart of the study cohort. FLLs, focal liver lesions; MRI, magnetic resonance imaging

From January 2017 to December 2022, all patients with pathologically confirmed CRC, whether identified via biopsy or surgical resection, who underwent Gd-EOB-DTPA-enhanced liver MRI during the initial workup for cancer staging were identified. Patients were excluded from the study if they had undergone surgical or ablation treatments prior to the Gd-EOB-DTPA-enhanced liver MRI or if they lacked subsequent imaging follow-up. Figure 1 presents a flow diagram that outlines the inclusion and exclusion criteria for patients in the study.

The final cohort consisted of 94 patients, including 70 males (74.5%) and 24 females (35.5%), with a mean age of 58.6 ± 11.7 years.

### MRI technique

The participants underwent contrast-enhanced liver MRI examinations using two MRI scanners: a 1.5-T scanner (MAGNETOM Aera) and a 3-T scanner (MAGNETOM Prisma), both manufactured by Siemens Healthineers in Erlangen, Germany. The imaging protocol comprised the following sequences: respiratory-triggered axial fat-suppressed TSE T2WI, breath-hold T1-weighted Dixon sequences in in-phase, opposed-phase, water, and fat configurations, DWI with b-values of 0, 50, 500, and 800 s/mm², and transverse breath-hold 3D fat-suppressed spoiled gradient-echo T1WI (3D GRE T1-WI).

After acquiring the pre-contrast sequences, Gd-EOB-DTPA (Primovist®, Bayer) was infused at a rate of 0.2 mL/kg over 1 mL/s, followed by a 20-mL saline flush using a power injector and bolus tracking. Standard late arterial, portal venous, and transitional phase images were then obtained. Hepatobiliary phase (HBP) images were acquired at least 20 min post-contrast injection, employing the identical fat-suppressed 3D GRE T1-WI sequences utilized in the pre-contrast scans. Arterial phase (AP) images were triggered automatically when the contrast media reached the ascending aorta. Portal venous phase (PVP) images were acquired 60 to 70 s after Gd-EOB-DTPA administration, and transitional phase (TP) images were obtained 150 to 180 s post-administration. A comprehensive overview of the MRI protocol is provided in Table 1.

**Table 1:** Details of the MRI acquisition protocol

Sequence	TR (ms)	TE (ms)	Slice thickness (mm)	Flip angle (°)	Bandwidth (Hz)	Matrix
Siemens Somatom Aera						
Respiratory-triggered T2-weighted turbo spin-echo (TSE)	4527	106	5.5	160	195	384 × 205
Respiratory-triggered T2-weighted BLADE	3000	110	5.5	156	365	320 × 320
BH T1-weighted VIBE DIXON	6.9	4.8/2.4	3.5	10	490	320 × 187
Diffusion-weighted imaging (*b* = 0, 50, 500, 800)	3200	56	5.5	90	1565	128 × 84
3D T1 FS GRE dynamic sequences (VIBE)	3.5	1.4	3.0	10	405	352 × 215
3D T1 FS GRE HBP (20 min)	3.5	1.4	3.0	10	405	352 × 215
Siemens Somatom Prisma						
Respiratory-triggered T2-weighted BLADE	4838.3	83	5.0	100	620	384 × 384
BH T1-weighted VIBE DIXON	4	2.5/1.3	3.0	9	1040	320 × 207
Diffusion-weighted imaging (*b* = 0, 50, 500, 800)	4900	60	5.0	90	2380	140 × 114
3D T1 FS GRE dynamic sequences (VIBE)	3.1	1.2	3.0	13	625	320 × 234
3D T1 FS GRE HBP (20 min)	3.1	1.2	3.0	13	620	352 × 229

Dynamic sequences consist of pre-contrast, late arterial, portal venous, and transitional phases

*TSE* turbo spin-echo, *BH* breath hold, *HBP* hepatobiliary phase, *GRE* gradient recall echo, *FS* fat saturation

### Three MRI protocols

**Fig. 2:**
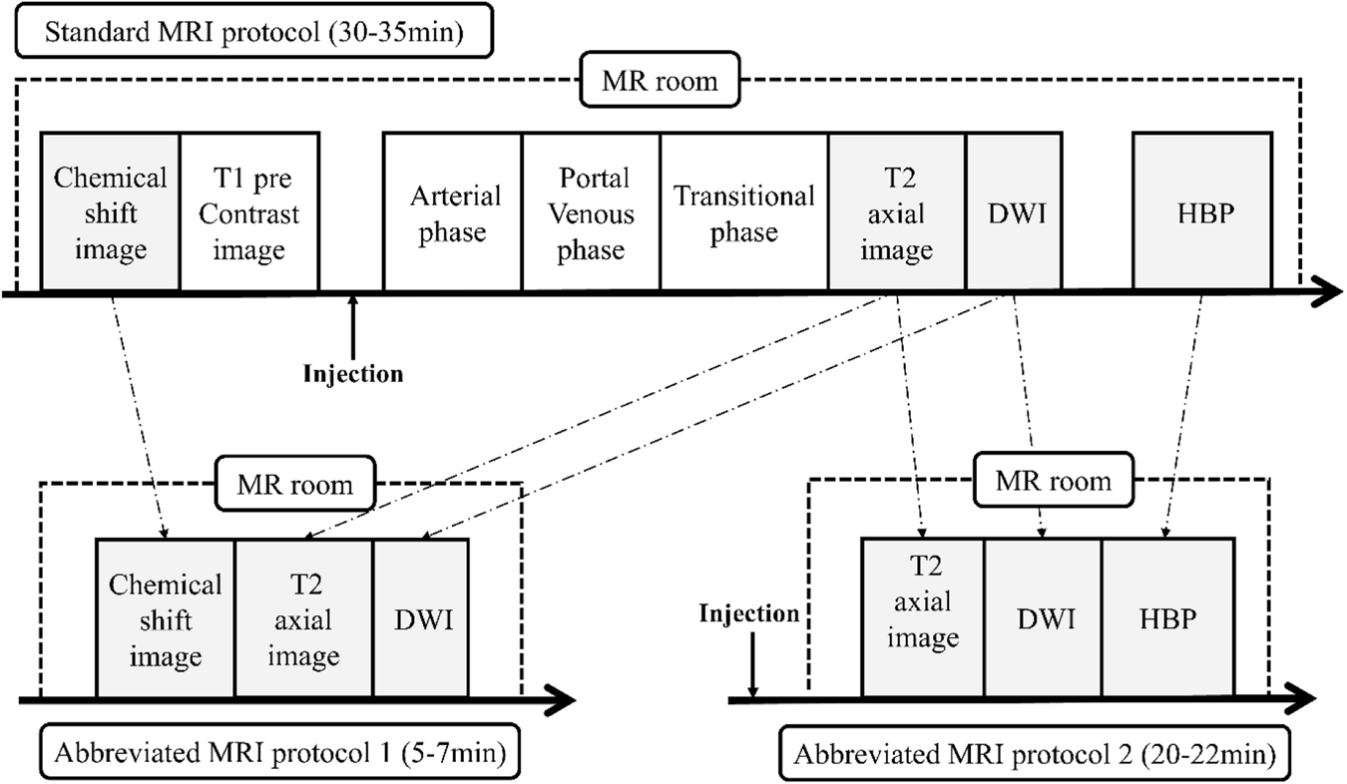
Schematic diagrams of the standard magnetic resonance imaging protocol (top diagram) and of the two types of simulated abbreviated magnetic resonance imaging protocols (bottom diagram). DWI, diffusion-weighted imaging; HBP, hepatobiliary phase; MR, magnetic resonance

Three distinct MR examination protocols (two abbreviated MRI (AMRI) protocols and one full MRI protocol) were analyzed in total: Protocol 1 comprised a non-contrast AMRI protocol, featuring axial T2 turbo spin-echo (TSE), axial T1-weighted Dixon images (in-phase, out-of-phase, water-weighted, and fat-weighted), diffusion-weighted imaging (DWI), and apparent diffusion coefficient (ADC) maps. Protocol 2 entailed an AMRI protocol including the hepatobiliary phase (HBP), consisting of axial T2 TSE, axial HBP images acquired 15–20 min post-gadoxetic acid injection, DWI, and ADC images. Protocol 3 encompassed all sequences of the standard gadoxetic acid-enhanced MRI protocol (Fig. 2).

At our institution, the standard gadoxetic acid-enhanced MRI protocol (Protocol 3) has a total scan acquisition time of approximately 30–35 min. The respiratory-triggered T2WI sequence takes about 2–5 min, the respiratory-triggered DWI sequence takes approximately 2–3 min, and a single breath-hold T1 Dixon sequence requires about 13–17 s. Consequently, the non-contrast abbreviated MRI protocol (Protocol 1) has a total acquisition time of approximately 5–7 min.

For the abbreviated MRI protocol including the hepatobiliary phase (HBP) (Protocol 2), according to current expert consensus, the recommended HBP scan time is 20 min [21]. Therefore, we assume that patients first receive manual contrast agent administration outside the scanner room and wait for approximately 15 min. They then enter the scanner room for a series of sequences: DWI, T2WI, and HBP. The 6-min scan time is included within the 20-min waiting period, as DWI and T2WI can be completed before the HBP imaging. The total scan time for this protocol, including DWI, T2WI, and HBP, is approximately 5–7 min. However, including the waiting period for the contrast agent to reach the HBP, the total time required for the patient is about 20–22 min.

### Image analysis

All MR examinations were compiled and anonymized by a radiologist (H.R.D.) not involved in the readings. Two radiologists (C.Y. and K.L.), each with 26 and 18 years of experience, respectively, in hepatic tumor imaging, retrospectively and independently reviewed all the examinations. The readers were blinded to previous radiological findings and pathological results but were aware that the patients had CRC, indicating a heightened likelihood of metastasis. Three protocols were read in sequence—first Protocol 1, then Protocol 2, and finally Protocol 3—with a 4-week interval between each to mitigate recall bias.

**Fig. 3:**
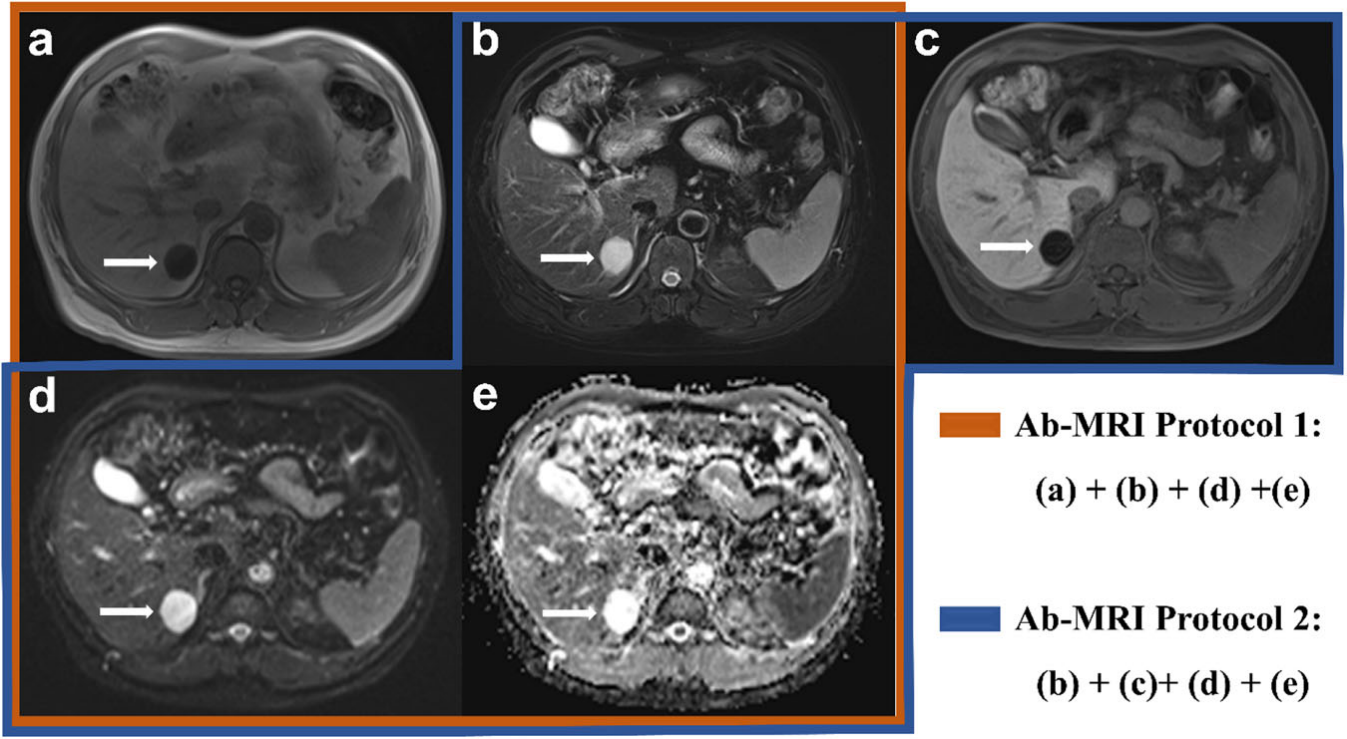
Display of two abbreviated MRI protocols in a 47-year-old male patient. **a** Dixon T1-weighted images (in-phase), (**b**) turbo spin-echo T2-weighted image, (**c**) hepatobiliary phase (HBP), (**d**) high b-value diffusion-weighted image (DWI), and (**e**) apparent diffusion coefficient (ADC) map. In segment VI, a lesion (arrow) was visualized that was characterized by low signal intensity on T1-weighted imaging, contrasting with elevated signal intensities on both DWI and T2-weighted sequences, coupled with a markedly high ADC value. Notably, this lesion showed marked hypointensity on HBP. Consistently, the lesion was assigned a consensus grade of 1 by two independent readers using two abbreviated MRI protocols, correlating with a diagnosis of hepatic cyst

**Fig. 4:**
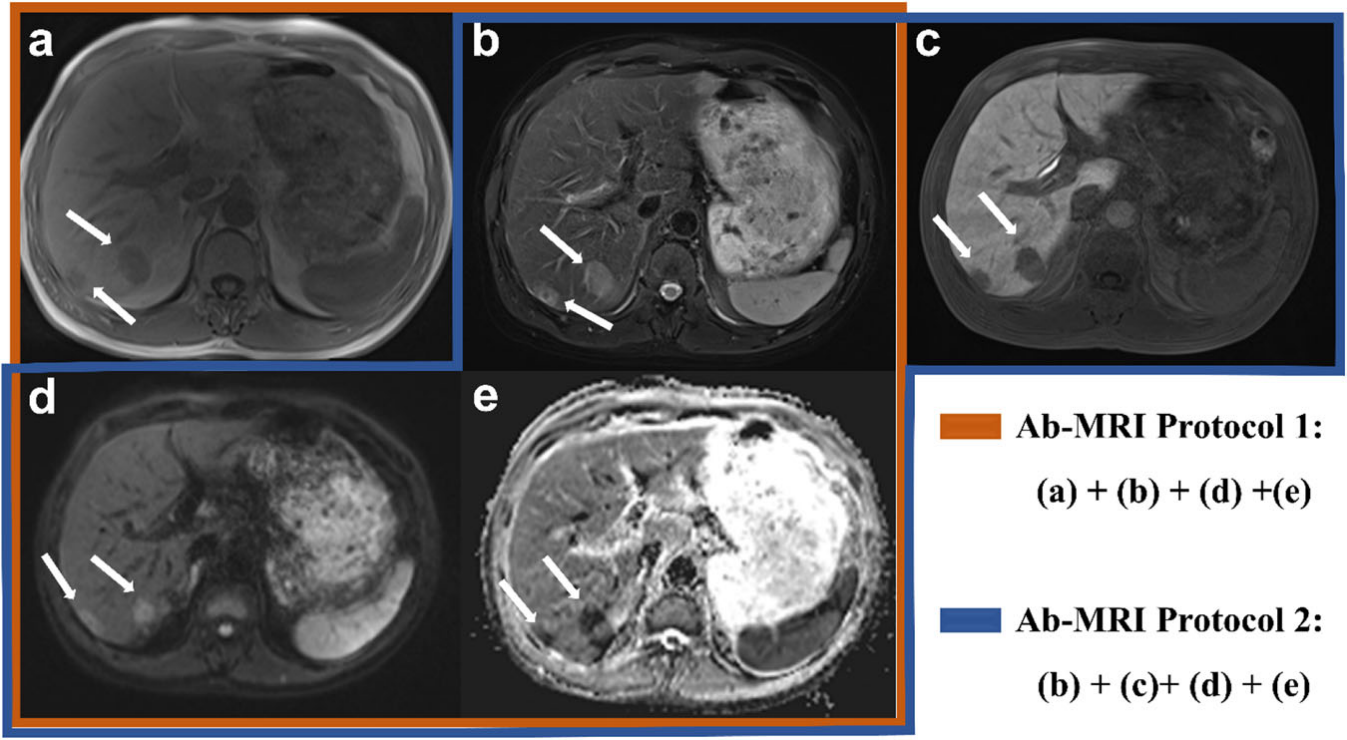
Display of two abbreviated MRI protocols in a 57-year-old male patient. **a** Dixon T1-weighted images (in-phase), (**b**) turbo spin-echo T2-weighted image, (**c**) hepatobiliary phase (HBP), (**d**) high b-value diffusion-weighted image (DWI), and (**e**) apparent diffusion coefficient (ADC) map. Segment VII revealed two lesions (indicated by arrows), displaying low T1 signal, high signals on DWI and T2, reduced ADC values, and notable HBP hypointensity. Consensus between two readers classified them as score 5 under both assessment protocols, correlating with a diagnosis of hepatic metastases

For each protocol set, the readers documented the number of all liver lesions, their segmental location according to the Couinaud classification system [22], and the lesion sizes. Each lesion was annotated individually. The readers were instructed to assess the likelihood of each lesion being a metastasis using a 5-point Likert scale, ranging from 1 (definitely benign) to 5 (definitely malignant), to evaluate the malignant potential of every detected abnormality (Figs. 3 and 4).

### Standard of reference

The reference standard aimed to categorize lesions as “metastatic” or “non-metastatic,” rather than to establish definitive diagnoses for all non-metastatic lesions.

Considering that the majority of patients had multiple metastatic tumors, and not all metastases in each patient were surgically resected for pathological biopsy, the pathological confirmation of part of the metastatic tumors and part of the benign lesions was obtained through the analysis of surgically resected specimens. For the remaining metastases and benign lesions, a multidisciplinary team, incorporating clinical information and evaluating morphological characteristics, size, and vascular patterns of the lesions, ascertained their benign nature following a minimum of 1 year of regular imaging surveillance, which entailed MRI or CT every 3 months.

### MRI cost analysis

Total costs for each MRI protocol were derived by considering both technical and professional components according to Medicare reimbursement standards. Given that the abbreviated protocols involve fewer sequences and shorter acquisition times, while the time and expertise required for image interpretation remain similar to those for standard contrast-enhanced MRI (CE-MRI), the cost of abbreviated MRI (AMRI) was estimated by maintaining the professional component cost unchanged and reducing the technical component cost by 50% [23].

The standard CE-MRI (HCPCS code 74182) served as the baseline with a total cost of $527.84. This cost included a technical component of $429.73 and a professional component of $98.11. For Ab-MRI 2, the professional component remained at $98.11, and the technical component was conservatively reduced by 50% from the standard CE-MRI technical component, resulting in a total cost of $312.98.

The cost of non-enhanced Ab-MRI 1 was based on the Medicare reimbursement for abdominal MRI without contrast (HCPCS code 74181). The technical component was reduced by 50% to $151.49, while the professional component remained at $81.88, resulting in a total cost of $233.37.

Additionally, we evaluated the number of follow-up examinations for each patient in the cohort, excluding the initial Gd-EOB-DTPA-enhanced MRI. Assuming that all patients underwent AMRI-M for surveillance, the total number of follow-up MRIs for the entire cohort was calculated. The total cost savings compared to using standard CE-MRI for the entire follow-up regimen was then estimated.

### Statistical analysis

Continuous variables were presented as mean ± standard deviation (SD). Categorical variables were summarized as counts and percentages. Continuous variables were tested for normal distribution using the Shapiro–Wilk test. Normally distributed variables were analyzed using Student’s *t*-test, while non-normally distributed variables were analyzed using the Mann–Whitney U test.

To assess the performance of the MR imaging program, two scores are created by combining lesions:

“Characterization Score A”: when the score is 4 or 5, the lesion is considered malignant (i.e., from “probably malignant” to “definitely malignant”)

“Characterization score B”: on a scale of 3 to 5, the lesion is considered to be malignant (i.e., from “uncertain” to “definitely malignant”).

Sensitivity, specificity, positive predictive value (PPV), negative predictive value (NPV), and accuracy per lesion for the three imaging protocols were determined, accompanied by their respective 95% confidence intervals (95% CI).

Indeterminate lesions were analyzed separately using Chi-square tests or Fisher’s exact tests. Lesion detection rates were compared using McNemar’s Chi-squared test, and interobserver concordance was assessed using Cohen’s kappa coefficients [24]. The diagnostic performance of each protocol was evaluated using ROC curve analysis, with AUCs calculated and compared using a univariate *z*-score test. Additionally, comparisons were made using the DeLong method. A bilateral *p*-value of less than 0.05 was considered statistically significant. All statistical analyses were conducted using the SPSS software, version 26.0.

## Results

### Patient demographics and lesion characteristics

Patient demographics and tumor characteristics are summarized in Table 2. The study cohort consisted of 94 patients with a mean age of 58.6 ± 11.7 years, comprising 70 males (74.5%) and 24 females (25.5%). Among these, 55 patients (58.5%) had colon cancer and 39 (41.5%) had rectal cancer. A total of 56 patients (59.6%) proceeded to undergo surgical resection of liver metastases.

**Table 2:** Patient and tumor characteristics

Patients	*N* = 94
Men	70 (74.5%)
Women	24 (25.5%)
Age: mean ± SD (years)	58.6 ± 11.7
Location of CRC	
Colon	55 (58.5%)
Rectum	39 (41.5%)
Surgical resection of liver metastases	
Yes	56 (59.6%)
No	38 (40.4%)
Total number of lesions	
Metastases	287 (68.0%)
Benign lesions	135 (32.0%)
Size: mean ± SD (mm)	12.3 ± 11
< 10	246 (58.3%)
≥ 10	176 (41.7%)
Proof (per lesion)	
Surgery	154 (36.5%)
Biopsy	7 (1.7%)
Imaging follow-up	261 (61.8%)

*SD* standard deviation, *CRC* colorectal cancer

A collective of 422 lesions were identified among the patients, with 287 confirmed as metastatic (68.0%) based on the reference standard. The mean lesion diameter was measured at 12.3 ± 11 mm. Diagnosis for 161 lesions (38.2%) was established through pathological examination, while the status of the other 261 lesions (61.8%) was determined via subsequent clinical and imaging follow-up.

### Lesion detection rates and scoring

Reader 1 detected 378 (89.6%) of the 422 lesions using Ab-MRI protocol 1, including 267 (93.0%) metastases and 111 (82.2%) benign lesions. Using Ab-MRI protocol 2, Reader 1 recognized 381 (90.3%) lesions, with 269 (93.7%) metastases and 112 (83.0%) benign lesions. Reader 2 detected 378 (89.6%) lesions, including 268 (93.4%) metastases and 110 (81.5%) benign lesions. With the Standard MRI protocol, Reader 1 identified 384 (91.0%) lesions, comprising 270 (94.1%) metastases and 114 (84.4%) benign lesions. Reader 2 detected 381 (90.3%) lesions, including 269 (93.7%) metastases and 112 (83.0%) benign lesions.

Neither reader observed statistically significant variations among the three protocols for all lesions or exclusively for metastases (*p*-values > 0.05 for all comparisons).

Detailed lesion scoring by both readers for each protocol is presented in Table 3.

**Table 3:** Lesion scoring among detected liver lesions

	Reader 1			Reader 2		
	Metastases	Benign lesions	Total	Metastases	Benign lesions	Total
Protocol 1						
Certainly benign	5 (1.9%)	83 (74.8%)	88 (23.3%)	2 (0.8%)	65 (59.1%)	67 (17.8%)
Probably benign	8 (3.0%)	11 (9.9%)	19 (5.0%)	3 (1.1%)	27 (24.5%)	30 (8.0%)
Indeterminate	7 (2.6%)	3 (2.7%)	10 (2.6%)	24 (9.0%)	10 (9.1%)	34 (9.0%)
Probably malignant	17 (6.4%)	5 (4.5%)	22 (5.8%)	31 (11.7%)	4 (3.6%)	35 (9.3%)
Certainly malignant	230 (86.1%)	9 (8.1%)	239 (63.2%)	206 (77.4%)	4 (3.6%)	210 (55.9%)
Total	267 (100.0%)	111 (100.0%)	378 (100.0%)	266 (100.0%)	110 (100.0%)	376 (100.0%)
Protocol 2						
Certainly benign	4 (1.5%)	83 (74.1%)	87 (22.8%)	5 (1.9%)	59 (53.6%)	64 (16.9%)
Probably benign	5 (1.9%)	14 (12.5%)	19 (5.0%)	3 (1.1%)	39 (35.5%)	42 (11.1%)
Indeterminate	4 (1.5%)	2 (1.8%)	6 (1.6%)	18 (6.7%)	2 (1.8%)	20 (5.3%)
Probably malignant	26 (9.7%)	7 (6.3%)	33 (8.7%)	58 (21.6%)	7 (6.4%)	65 (17.2%)
Certainly malignant	230 (85.5%)	6 (5.4%)	236 (61.9%)	184 (68.7%)	3 (2.7%)	187 (49.5%)
Total	269 (100.0%)	112 (100.0%)	381 (100.0%)	268 (100.0%)	110 (100.0%)	378 (100.0%)
Protocol 3						
Certainly benign	4 (1.5%)	92 (80.7%)	96 (25.0%)	4 (1.5%)	76 (67.9%)	80 (21.0%)
Probably benign	5 (1.9%)	11 (9.6%)	16 (4.2%)	4 (1.5%)	25 (22.3%)	29 (7.6%)
Indeterminate	3 (1.1%)	2 (1.8%)	5 (1.3%)	8 (3.0%)	4 (3.6%)	12 (3.1%)
Probably malignant	16 (5.9%)	5 (4.4%)	21 (5.5%)	25 (9.3%)	5 (4.5%)	30 (7.9%)
Certainly malignant	242 (89.6%)	4 (3.5%)	246 (64.1%)	228 (84.8%)	2 (1.8%)	231 (60.6%)
Total	270 (100.0%)	114 (100.0%)	384 (100.0%)	269 (100.0%)	112 (100.0%)	381 (100.0%)

### Diagnostic uncertainty

To evaluate diagnostic uncertainty, the frequency of indeterminate lesions was analyzed for each protocol. Protocol 1 had the highest frequency of indeterminate lesions for both readers, with Reader 1 identifying 10 out of 378 lesions (2.6%) and Reader 2 identifying 34 out of 376 lesions (9.0%). Protocol 3 had the lowest frequency of indeterminate lesions, with Reader 1 identifying 5 out of 384 lesions (1.3%) and Reader 2 identifying 12 out of 381 lesions (3.1%). Statistical analysis showed significant differences between protocols, with Reader 1 observing significant differences between Protocol 1 and Protocol 2 (χ² = 4.27, *p* = 0.039) and between Protocol 1 and Protocol 3 (χ² = 7.5, *p* = 0.006), while the difference between Protocol 2 and Protocol 3 was not significant (χ² = 0.367, *p* = 0.545). For Reader 2, significant differences were observed between Protocol 1 and Protocol 2 (χ² = 15.56, *p* ≈ 0.0001), between Protocol 1 and Protocol 3 (χ² = 54.57, *p* < 0.0001), and between Protocol 2 and Protocol 3 (χ² = 8.53, *p* = 0.0036).

### Diagnostic performance

The diagnostic performance of each protocol is summarized in Table 4. For characterization score A, Protocol 3 showed the highest AUC values for both Reader 1 (0.938 [0.906–0.970]) and Reader 2 (0.939 [0.908–0.970]). For characterization score B, Protocol 3 also demonstrated the highest AUC values for both Reader 1 (0.935 [0.901–0.969]) and Reader 2 (0.936 [0.902–0.970]).

**Table 4:** Comparison of diagnostic performance of three protocols according to the characterization scores

	Reader 1			Reader 2		
	Protocol 1	Protocol 2	Protocol 3	Protocol 1	Protocol 2	Protocol 3
Characterization score A						
Sensitivity	92.5 (88.5–95.3)	95.2 (91.7–97.3)	95.6 (92.2–97.6)	89.1 (84.6–92.5)	90.3 (86.0–93.5)	94.1 (90.3–96.5)
Specificity	87.3 (79.4–92.7)	88.4 (80.6–93.4)	92.1 (85.1–96.1)	92.7 (85.7–96.6)	90.9 (83.5–95.3)	93.8 (87.1–97.2)
PPV	93.7 (90.0–96.2)	95.1 (91.7–97.3)	96.6 (93.5–98.4)	96.7 (93.4–98.5)	96.0 (92.6–98.0)	97.3 (94.3–98.8)
NPV	83.7 (75.5–89.7)	88.4 (80.6–93.4)	89.7 (82.4–94.4)	77.9 (69.6–84.5)	79.4 (71.1–85.9)	86.8 (79.1–92.0)
Accuracy	91.0 (87.6–93.6)	93.2 (90.0–95.4)	94.5 (91.6–96.5)	90.2 (86.6–92.9)	90.5 (87.0–93.2)	94.0 (91.0–96.1)
AUC	0.899 [0.859–0.940]	0.918 [0.880–0.955]	0.938 [0.906–0.970]	0.909 [0.874–0.945]	0.906 [0.869–0.943]	0.939 [0.908–0.970]
Characterization score B						
Sensitivity	95.1 (91.6–97.3)	96.7 (93.5–98.4)	96.7 (93.6–98.4)	98.1 (95.4–99.3)	97.0 (94.0–98.6)	97.0 (94.0–98.6)
Specificity	85.6 (77.4–91.3)	86.6 (78.6–92.1)	90.4 (83.0–94.9)	83.6 (75.1–89.9)	89.1 (81.4–94.0)	90.2 (82.7–94.8)
PPV	91.9 (87.2–95.1)	94.6 (91.0–96.8)	96.0 (92.7–97.9)	93.6 (89.2–96.0)	95.6 (92.2–97.6)	96.0 (92.7–97.9)
NPV	87.9 (79.8–93.1)	91.5 (84.1–95.8)	92.0 (84.9–96.0)	94.9 (87.8–98.1)	92.5 (85.2–96.5)	92.7 (85.6–96.6)
Accuracy	92.1 (88.7–94.5)	93.7 (90.6–95.8)	94.8 (91.9–96.7)	93.9 (90.8–96.0)	94.7 (91.8–96.7)	95.0 (92.2–96.9)
AUC	0.899 [0.857–0.941]	0.916 [0.877–0.955]	0.935 [0.901–0.969]	0.909 [0.867–0.951]	0.931 [0.894–0.967]	0.936 [0.902–0.970]

*AUC* area under the receiver operating characteristic curve, *NPV/PPV* negative/positive predictive value

### Comparison of AUC values

**Fig. 5:**
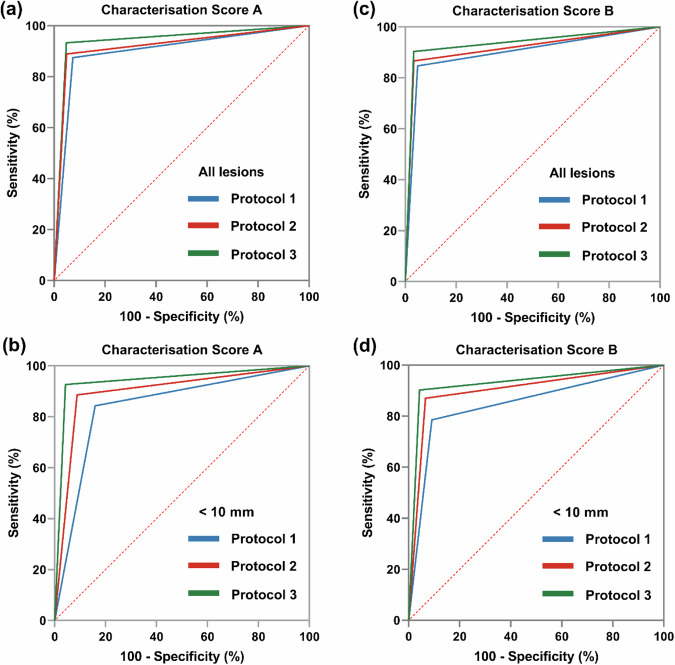
Comparative ROC analysis of three protocols by reader 1 using divergent characterization scores. **a** The AUC values and 95% CI of the three protocols based on characterization score A in all lesion groups were 0.899 (0.859–0.940), 0.918 (0.880–0.955) and 0.938 (0.906–0.970), respectively. **b** In lesions < 10 mm, the values were 0.842 (0.784–0.900), 0.899 (0.852–0.946), and 0.942 (0.906–0.978), respectively. **c** The AUC values and 95% CI of three protocols based on the characterization score B in all lesion groups were 0.899 (0.857–0.941), 0.916 (0.877–0.955) and 0.935 (0.901–0.969), respectively. **d** For lesions < 10 mm, the values were 0.847 (0.791–0.903), 0.902 (0.850–0.948) and 0.930 (0.891–0.969), respectively. AUC, area under the receiver operating characteristic curve; 95% CI, 95% confidence intervals

A subgroup analysis, based on per-lesion scoring from both readers, compared the AUCs of the three protocols for all lesions (*n* = 422) and those < 10 mm (*n* = 246) (Table 5 and Fig. 5). For Characterization Score A, Protocol 3 showed higher AUC values compared to Protocols 1 and 2, though the differences were not statistically significant when compared to Protocol 1 (Reader 1: *z* = 1.48, *p* = 0.069; Reader 2: *z* = 1.24, *p* = 0.108). However, for lesions < 10 mm, Protocol 3’s accuracy was significantly better than Protocol 1 (Reader 1: *z* = 2.86, *p* = 0.002; Reader 2: *z* = 1.88, *p* = 0.030).

**Table 5:** Comparison of AUCs of three protocols in subgroup analysis according to the characterization scores

	All lesions			Lesions < 10 mm		
	Reader 1	Reader 2	k values	Reader 1	Reader 2	k values
Characterization score A						
Protocol 1	0.899 [0.859–0.940]	0.909 [0.874–0.945]	0.850	0.842 [0.784–0.900]	0.884 [0.832–0.936]	0.846
Protocol 2	0.918 [0.880–0.955]	0.906 [0.869–0.943]	0.859	0.899 [0.852–0.946]	0.907 [0.861–0.952]	0.873
Protocol 3	0.938 [0.906–0.970]	0.939 [0.908–0.970]	0.807	0.942 [0.906–0.978]	0.945 [0.909–0.980]	0.821
*z*/*p*-value (1 vs. 3)	1.48/0.069	1.24/0.108		2.86/0.002	1.88/0.030	
*z*/*p*-value (2 vs. 3)	0.81/0.209	1.33/0.092		1.30/0.096	1.30/0.096	
Characterization score B						
Protocol 1	0.899 [0.857–0.941]	0.909 [0.867–0.951]	0.904	0.847 [0.791–0.903]	0.884 [0.835–0.933]	0.885
Protocol 2	0.916 [0.877–0.955]	0.931 [0.894–0.967]	0.924	0.902 [0.850–0.948]	0.900 [0.854–0.947]	0.853
Protocol 3	0.935 [0.901–0.969]	0.936 [0.902–0.970]	0.877	0.930 [0.891–0.969]	0.941 [0.906–0.976]	0.896
*z*/*p*-value (1 vs. 3)	1.33/0.092	1.00/0.158		2.41/0.008	1.85/0.032	
*z*/*p*-value (2 vs. 3)	0.725/0.236	0.202/0.421		0.92/0.179	1.37/0.085	

*AUC* area under the receiver operating characteristic curve

For Characterization Score B, Protocol 3 also showed higher AUC values, but the differences were not statistically significant when compared to Protocol 1 (Reader 1: *z* = 1.33, *p* = 0.092; Reader 2: *z* = 1.00, *p* = 0.158). For lesions < 10 mm, Protocol 3 was more accurate than Protocol 1 (Reader 1: *z* = 2.41, *p* = 0.008; Reader 2: *z* = 1.85, *p* = 0.032). Comparisons with Protocol 2 did not reveal statistically significant differences, with both readers reporting *p*-values above 0.05.

### Interobserver agreement

The degree of inter-reader concordance in lesion classification is presented in Table 5. Specifically, the kappa statistics for tumors jointly identified by both readers under Protocol 1 ranged from 0.850 to 0.904, indicative of remarkable consensus and classified as excellent agreement. Similarly, Protocol 2 yielded an outstanding inter-reader agreement, as evidenced by kappa values within the range of 0.859 to 0.924. In examining Protocol 3, the kappa coefficients demonstrated a range of 0.807 to 0.877, thereby substantiating the continued presence of a notable consensus in the assessments provided by the two readers.

### Estimated cost savings

The average number of follow-up MRIs per patient was approximately 5, with a standard deviation of 4. For the standard CE-MRI, the average cost per patient was $2639.20, resulting in a total cost of $248,476.80 for 94 patients.

For the non-enhanced Ab-MRI 1, the average cost per patient was $1166.85, leading to a total cost of $110,007.90 for 94 patients. The cost savings per patient compared to CE-MRI were $1472.35, resulting in a total savings of $138,455.90 for the entire cohort.

For Ab-MRI 2, the average cost per patient was $1564.90, leading to a total cost of $147,170.60 for 94 patients. The cost savings per patient compared to CE-MRI were $1074.30, resulting in a total savings of $100,780.20 for the entire cohort.

## Discussion

The findings of our study indicate that both the non-enhanced Ab-MRI protocol (Protocol 1) and the gadoxetic acid-enhanced Ab-MRI protocol (Protocol 2) exhibit high lesion detection capabilities for CRLM, matching the standard MRI protocol’s performance. This equality in diagnostic capability is observed for both the detection and characterization of CRLM, which may be attributed to the incorporation of diffusion-weighted images and T2-weighted sequences in all protocols. Although there are variations in the appearance of CRLM on DWI, DWI is essential in non-enhanced liver imaging due to its high sensitivity to malignant lesions [25, 26], though it lacks specificity for CRLM. On T2-weighted images, cysts and hemangiomas show pronounced high signal intensity, whereas liver metastases display only mild hyperintensity [27]. Additionally, T2WI clearly depicts lesion margins, internal structures, and concomitant inflammatory changes within the liver [28].

For lesions smaller than 10 mm, Protocol 2 of Ab-MRI demonstrated superior diagnostic performance compared to Protocol 1. This advantage is attributed to the inclusion of gadoxetic acid-enhanced hepatobiliary phase images. Evidence indicates that HBP enhances the detection of early-stage hepatic malignancies, especially those under 1 cm [29–32]. Similarly, Protocol 1 exhibited more diagnostic uncertainty compared to Protocol 2 and Protocol 3, likely due to the lack of gadoxetic acid-enhanced hepatobiliary phase images. Additionally, reader experience and lesion size are factors that can influence diagnostic uncertainty. Therefore, in a clinical screening scenario employing non-enhanced Ab-MRI 1, it is recommended to commence with a baseline contrast-enhanced liver MRI for comprehensive lesion characterization, followed by consecutive semiannual afferent non-enhanced Ab-MRI sessions for comparative monitoring. It is anticipated that this approach will enhance reader confidence and, in turn, minimize diagnostic uncertainty and improve overall diagnostic accuracy.

Several studies support the use of “abbreviated MRI” protocols, which employ T2WI, DWI, and HBP after gadoxetic acid administration. These protocols omit non-contrast T1WI and dynamic contrast-enhanced imaging, allowing for faster scanning. For example, Canellas et al [16] evaluated a similar abbreviated protocol, showing high sensitivity (> 90%) and excellent inter-reader agreement (κ = 0.91). They also noted a significant cost reduction (59% of standard MRI cost) without compromising diagnostic yield. In accordance with these findings, Ghorra et al [17] confirmed similar performance for CRLM detection using a comparable short MRI protocol. These findings align with our study results.

Advancements in functional sequences like DWI have led to a reconsideration of the need for contrast agents in surveillance scenarios. In the context of surveillance, the primary responsibility of the radiologist is to identify residual metastases and assess resectability. Given that all lesions are known at this stage, administering contrast agents may be unnecessary. Our findings are in accordance with those of Whang et al [33], who demonstrated comparable diagnostic performance between non-enhanced MRI and abbreviated MRI with gadoterate for early hepatocellular carcinoma detection. Similarly, Granata et al [34] also found a simplified MRI protocol, including DWI and T2-weighted fat-suppressed sequences, effective for detecting colorectal liver metastases. The results of their study corroborated the efficacy of the non-enhanced Ab-MRI protocol in detecting liver metastases to a level comparable to that of the enhanced MRI.

Moreover, non-enhanced MRI protocols present distinct advantages over gadoterate-containing “abbreviated MRI” protocols for surveillance purposes. It is important to note that non-enhanced MRI should be considered a monitoring instrument rather than a definitive diagnostic tool. While highly sensitive and accurate, non-enhanced MRI should be used for monitoring rather than initial staging. Initial staging requires detailed lesion characterization, especially for sub-centimeter lesions. Therefore, standard CE-MRI should be used for initial staging. Moreover, a surveillance test should be evaluated not only in terms of its accuracy but also in terms of its affordability, compliance, and cost-effectiveness. Non-enhanced MRI offers shorter scan times, reduced costs, and eliminates venous catheterization and associated complications. Furthermore, the use of a ‘curtailed MRI protocol’ involving gadolinium-based extracellular agents for CRLM monitoring presents another potential disadvantage. Although there is no evidence that gadolinium retention causes harm in individuals with normal kidney function, recurrent dosing should be avoided due to the heightened risk of gadolinium retention [35]. Given the necessity of periodic repetitive examinations for CRLM surveillance, we propose non-enhanced Ab-MRI protocol as a viable alternative to contrast-enhanced MRI protocols.

However, we should acknowledge that the proposal of non-enhanced MRI as a potential alternative for monitoring CRLM is not without limitations. In our investigation, non-enhanced MRI was found to be unable to detect lesions located at the dome of the diaphragm and beneath the capsule. This is potentially due to susceptibility and aliasing artifacts that plague DWI in the upper abdomen. These artifacts, which are associated with the presence of gas in the nearby intestine and physiological motion, can respectively obscure lesions situated along the upper margin or in proximity to the diaphragm [36]. The enhanced detectability offered by HBP images may offset these limitations. However, in light of our recommendation for initial staging with standard CE-MRI to comprehensively characterize all pre-existing lesions, these limitations are deemed acceptable within the context of surveillance practices.

Limitations of our study include its retrospective design and limited cohort size. Prospective studies with larger populations are needed to confirm these observations. Not all lesions were histopathologically confirmed, but this is common in clinical management of CRLM, where diagnoses often rely on imaging findings. Surgical intervention decisions were based solely on conventional MRI, limiting the evaluation of the rapid protocol’s effectiveness in surgical cases. Finally, the study focused on hepatic tumors, excluding other metastatic sites.

## Conclusion

The non-enhanced abbreviated MRI protocol demonstrates equivalent diagnostic efficacy to gadoxetic acid-enhanced abbreviated MRI protocol in identifying CRLM in affected patients. Our proposed Ab-MRI approach may be considered a viable alternative to conventional MRI regimens for the surveillance of colorectal liver metastases.

## Data Availability

The datasets generated or analyzed during this study are available from the corresponding author upon reasonable request.

## Abbreviations

Ab-MRI: Abbreviated magnetic resonance imaging
AUC: Area under the curve
CRC: Colorectal cancer
CRLM: Colorectal liver metastases
Gd-EOB-DTPA: Gadolinium ethoxybenzyl diethylenetriamine pentaacetic acid
HBP: Hepatobiliary phase
ROC: Receiver operating characteristic (curves)
T2WI: T2-weighted imaging
TSE: Turbo spin echo
